# NURR1 Is Differentially Expressed in Breast Cancer According to Patient Racial Identity and Tumor Subtype

**DOI:** 10.3390/biomedinformatics2040045

**Published:** 2022-12-07

**Authors:** Shahensha Shaik, Ha’reanna Campbell, Christopher Williams

**Affiliations:** Division of Basic Pharmaceutical Sciences, College of Pharmacy, Xavier University of Louisiana, 1 Drexel Dr, New Orleans, LA 70125, USA

**Keywords:** NURR1, breast carcinoma, estrogen receptor, Oncotype Dx biomarkers, luminal A

## Abstract

Breast carcinoma (BCa) remains the second most common cause of cancer-related death among American women. Whereas estrogen receptor (ER) expression is typically regarded as a favorable prognostic indicator, a significant proportion of ER(+) patients still experience either de novo or acquired endocrine resistance. Previously, we have shown that the loss of orphan nuclear receptor NURR1 expression is associated with neoplastic transformation of the breast epithelium and shorter relapse-free survival (RFS) among systemically treated breast cancer (BCa) patients. Here, we further ascertain the prognostic value of NURR1 in BCa, and its differential expression among Black and White female BCa patients. We assessed the expression of NURR1 mRNA in BCa patients using the Cancer Genome Atlas (TGCA) and compared the occurrence of basal-like cancer and luminal A breast cancer subtypes. Expression levels were further stratified according to racial identity of the patient. We next assessed the correlation of NURR1 expression with Oncotype DX prognostic markers, and the association of NURR1 expression with relapse free survival in patients treated with endocrine therapy. Our study shows that NURR1 mRNA expression is differentially correlated with luminal A vs. basal-like cancer BCa and is predictive of poor relapse-free survival, confirming a similar trend observed in our previous studies using microarray data. NURR1 expression was positively correlated with expression of Oncotype DX biomarkers associated with estrogen responsiveness, while being inversely correlated with biomarkers associated with cell proliferation. Furthermore, we observed that NURR1 expression was positively associated with greater relapse-free survival at 5 years among patients treated with endocrine therapy. Interestingly, we found that among Black women with luminal A BCa, NURR1 expression was repressed in comparison to White women with the same subtype.

## Simple Summary

Breast cancer (BCa) is one of the leading causes of cancer death in women. The majority of breast cancers are estrogen receptor positive ER(+) and initially respond well to endocrine therapy. However, some patients with ER(+) BCa do not respond to endocrine therapy resulting in poor survival outcomes. Analyzing the Cancer Genome Atlas datasets, we found that patients with low expression of NURR1 gene with ER(+) BCa have poor survivorship in response to treatment. This low NURR1 expression is predominantly found in Black patients compared to other population groups leading to the disparity in ER(+) BCa. NURR1 expression can serve as a prognostic biomarker in identifying ER(+) BCa patients that may benefit from early, aggressive treatment strategies.

## 1. Introduction

In the United States, breast cancer is the most commonly diagnosed and major cause of cancer mortality among women after lung cancer [1]. According to the American Cancer Society, the mortality rate among Black women was 40% higher than their White counterparts during the period of 2013–2017 despite similar or slightly lower levels of breast cancer incidence [2]. A series of studies have shown that Black women have a greater risk of developing basal-like cancer and/or triple negative breast cancers, which are more difficult to treat with targeted agents and are typically regarded as a more advanced cancers [3–5]. However, studies show that Black BCa patients with molecular tumor subtypes other than basal-like cancer-like (i.e., luminal A and luminal B) have greater mortality compared to their counterparts as well [6,7]. A recent clinical study identified significant differences in differentially expressed genes associated with the luminal tumors between Black and White BCa patients [8]. The deadly combination of these molecular and biochemical predispositions along with several socioeconomic factors such as lower income, lack of proper health insurance, inaccessibility to advanced healthcare facilities for early diagnosis, obesity and its related co-morbidities, all contribute to the increased risk of mortality endured by Black BCa patients [5].

Our intent is to determine if there exist specific biomarkers that can further stratify patients beyond the current molecular and histological subtypes to better assess the BCa-related mortality risk, and if those markers are relevant to Black patients who have historically been excluded from clinical studies. Currently, the “gold standard” for assessing recurrence risk for patients with an ER(+) and lymph node-negative BCa diagnosis is the Oncotype DX genomic test [9–12]. In this test, the expression of 21 genes (shown in Table 1) that can likely affect the BCa response to a given BCa treatment is analyzed by RT-PCR [13,14]. The assay assigns a recurrence score (RS) that serves as a clinical guide for the treatment of BCa [13,14]. For example, RS above 31 indicates high cancer risk recurrence and treating such patients with chemotherapy will likely be beneficial as the overall outcome outweighs the side effects [13,14].

NURR1 (NR4A2) is a member of the NR4A family of orphan nuclear receptors (NR4A1, NR4A2, and NR4A3) [15,16]. NR4A receptors have been shown to play important roles in multiple biological functions, including inflammation, metabolism, cancer, and neuronal differentiation [15,16]. Designated as orphan receptors whose physiologic function appears to lack dependence on endogenous ligands, NR4A receptors are regulated primarily at the level of transcription and posttranslational modification events, such as phosphorylation. Until recently, NR4A receptors were considered “true orphans”, due to the existence of bulky aliphatic amino acid side chains which, according to crystal structures, fill the putative ligand binding cavity thereby precluding canonical ligand binding. However, several ligands have been identified in recent years, which either bind to atypical sites outside of the binding pocket, or stabilize resonant states within the ligand binding pocket, thereby stabilizing specific receptor conformations [16].

NURR1 appears to play divergent roles in cancer progression [17]. In breast biopsy samples, NURR1 was highly expressed in non-cancerous breast epithelium [18]. Lower NURR1 levels were evident in transformed tissue, suggesting that NURR1 silencing is permissive of oncogenic transformation [18]. Interestingly however, we showed that repression of NURR1 using shRNA in breast cancer xenograft models resulted in decreased tumor growth [18]. As such it seems that the role of NURR1 in breast cancer progression is highly nuanced and context dependent.

In this study, we sought to further characterize the prognostic significance of NURR1 expression in BCa, and whether a differential prognostic significance exists based on the population under study. Utilizing TCGA (the Cancer Genome Atlas) PanCancer Atlas data through the KMPlotter and CBioportal webtools, we assessed the expression of NURR1 in luminal A and basal-like cancer BCa, and among Black and White patients stratified by molecular subtype. Additionally, we compared the prognostic significance of NURR1 to that of the individual Oncotype DX RS component genes and assessed the correlation of NURR1 with better 5yr RFS rates among patients taking endocrine therapy. It is our assertion that NURR1 may function as a specific biomarker that may help to identify higher risk Luminal A BCa patients and contribute to reducing the disparity in RFS suffered by Black women with breast cancer.

## 2. Methods

### 2.1. Kaplan–Meier Survival Curves

The Kaplan Meier (KM) plotter identifies the association between mRNA expression and the relapse free survival of cancer patients. The KM-plotter PanCancer-RNA-Seq database has sample size of 1090 breast cancer patients from which the relapse-free survival (RFS) of the breast cancer patients with the NURR1 and the Oncotype DX panel gene expression in different racial groups was obtained [19,20]. We used KMPLOT (kmplot.org) to generate Kaplan-Meier survival curves for overall survival of BCa patients with high (upper quartile) or low (lower quartile) expression of NURR1 based on mRNA-seq data in the Cancer Genome Atlas (TGCA) PanCancer Atlas [19,20].

### 2.2. Analysis of Cancer Subtypes in Different Racial Groups

To identify the cancer subtypes in different racial groups we used CBioPortal.org [21,22]. The data was stratified in accordance with identification as Black or White. NR4A2 gene expression data (Z-score) for basal-like cancer and luminal A (LumA) subtypes for Black and White population was downloaded from PanCancer atlas in CBioportal. Furthermore, using CBioportal, we interrogated the same cohort of patient samples to determine the correlation of NURR1 verses that of Oncotype DX component genes to overall survival (OS) in Black BCa patients to determine if NURR1 is a comparable predictive factor for this cohort of patients.

### 2.3 . ROC Plotter Predictive Biomarker

ROC plotter is an online tool used to identify predictive biomarkers using transcriptomic data of a large set of breast cancer patients [23]. ROC plotter was used to determine the biomarker status of NURR1 expression in total population undergoing endocrine therapy with 5-year relapse free survival.

### 2.4. Statistical Analysis

**Figure 1.**
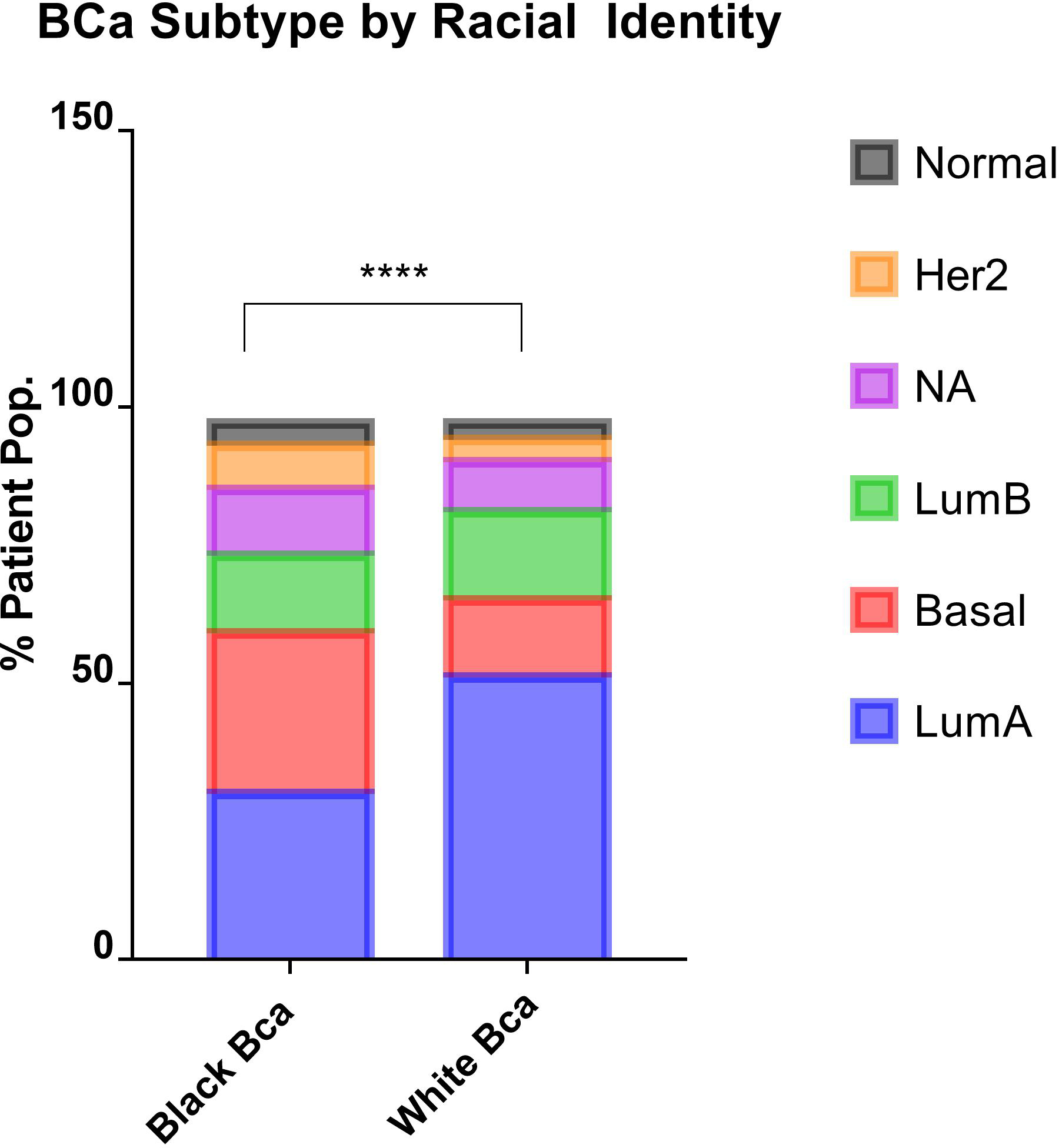
Basal cancer breast cancer occurrence is higher in Black patients. Comparison of the occurrence of the breast cancer subtypes between Black and White patients from TGCA PanCancer atlas database. The bar graph on the left shows the percentage of patient population in each BCa subtype. The number of patients obtained for this analysis is indicated in the table on the right. The overall statistical significance between the two groups was determined using chi-squared test, which resulted in the *p* value (****) < 0.0001 and the statistical difference between groups of same BCa subtype was determined using Fisher’s exact test, which is *p* value (****) < 0.0001 for LumA and basal while all others groups are found to be not significant.

The KM survival plots with number at risk, hazard ratio (HR), 95% confidence intervals (CI) and log-rank *p*-values were obtained using the KM plotter website. The *p*-values less 0.05 were considered as statistically significant. Two-tailed unpaired *t*-test for data sets with two groups and one-way ANOVA analysis was used for comparison of multiple datasets using Graphpad Prism software. For Figure 1, the statistical analysis was performed using chi-squared test followed by Fisher’s exact test.

## 3. Results

**Figure 2.**
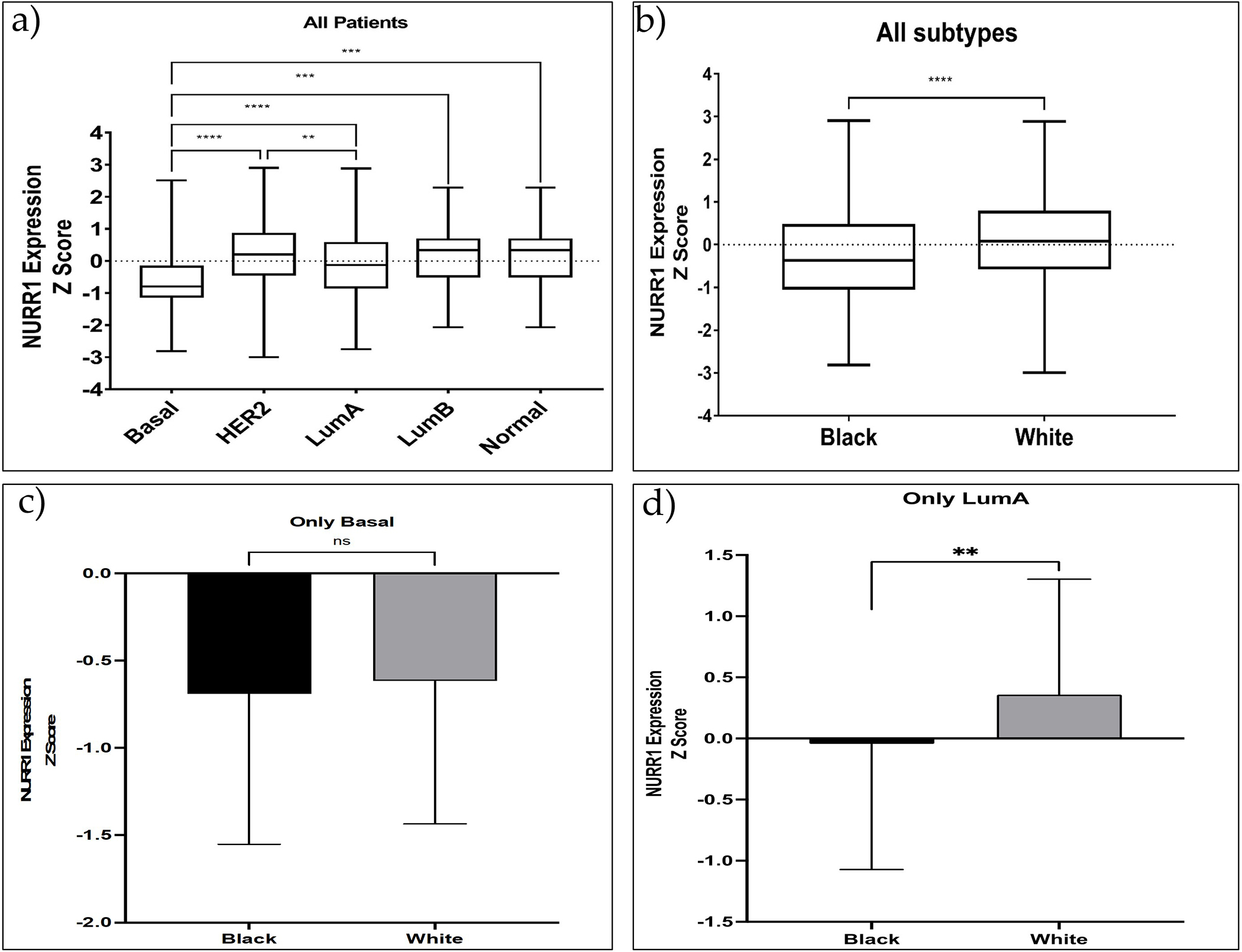
Expression levels of NURR1 in different BCa subtypes among Black and White BCa women. Overall expression of NURR1 (**a**) was found to be higher in LumA subtype and low in Basal-like cancer subtype in all BCa women; (**b**) was found to be low in Black BCa women than in other groups; (**c**) in Basal-like cancer subtype was found to be low in both Black and White groups; (**d**) was low especially in Black women with LumA subtype BCa. **** (*p* < 0.0001), *** (*p* = 0.0034), ** (*p* = 0.0189), ns: not significant.

The analysis of Carolina Breast Cancer Study by Carey et al., outlined the disproportionate incidence of basal-like breast cancer among Black women, demonstrating that 39% of tumors from premenopausal Black women presented with a basal-like cancer phenotype, as compared to 16% of non-Black patients [24]. We observed a similar phenomenon within the PanCancer Atlas cohort, with Black identity being associated with greater rates of basal-like cancer BCa phenotype compared to White patients (29% and 14% respectively). This is commensurate with a lower proportional incidence of luminal A cancer among Black patients 30.8% vs. 51.1% in White patients (Figure 1). Since NURR1 expression was found to be silenced in basal-like cancer BCa, we ascertained the expression of NURR1 among patients stratified according to racial identity (Figure 2).

Our previous studies have suggested that the expression of NURR1 may be associated with greater RFS in BCa patients undergoing systemic treatment [18]. Given that greater RFS is typically associated with luminal A BCa phenotypes, it stands to reason that the predictive value of NURR1 expression may be related to the molecular subtype of the tumor. As such we interrogated the TGCA PanCancer atlas for the expression of NURR1 (Z Scores) among BCa patients with luminal A (Z = −0.513), luminal B (Z = −0.9325), basal cancer (Z = −1.606), Her2 (Z = −1.139), and normal (Z = −0.6822) BCa (Figure 2). The expression of NURR1 mRNA was observed at lower levels in basal-like cancer whereas luminal A cancer has highest expression in comparison to all other subtypes (Figure 1). The statistical difference between Lum A vs. basal BCa and Her2 vs. basal was found to be of a greater significance (*p* < 0.0001) suggesting that LumA and Her2 cancers have overall higher expression than others (Figure 2a). Analysis of NURR1 expression across different race groups revealed that the Black BCa patients have lowest NURR1 expression (Z = −0.3158) than other groups with statistical significance between Black and White BCa groups (*p* < 0.0001) (Figure 2b). Therefore, we next compared the NURR1 expression in Black vs. White BCa groups, especially in basal and luminal A subtypes. In basal subtype NURR1 was lower in both groups with no statistical significance between them (Figure 2c). Interestingly, in LumA subtype NURR1 expression was found to be suppressed in the Black BCa group (Z = −0.043) compared to the White BCa group (Z = 0.358) with statistical significance (*p* value 0.034) (Figure 2d).

**Figure 3.**
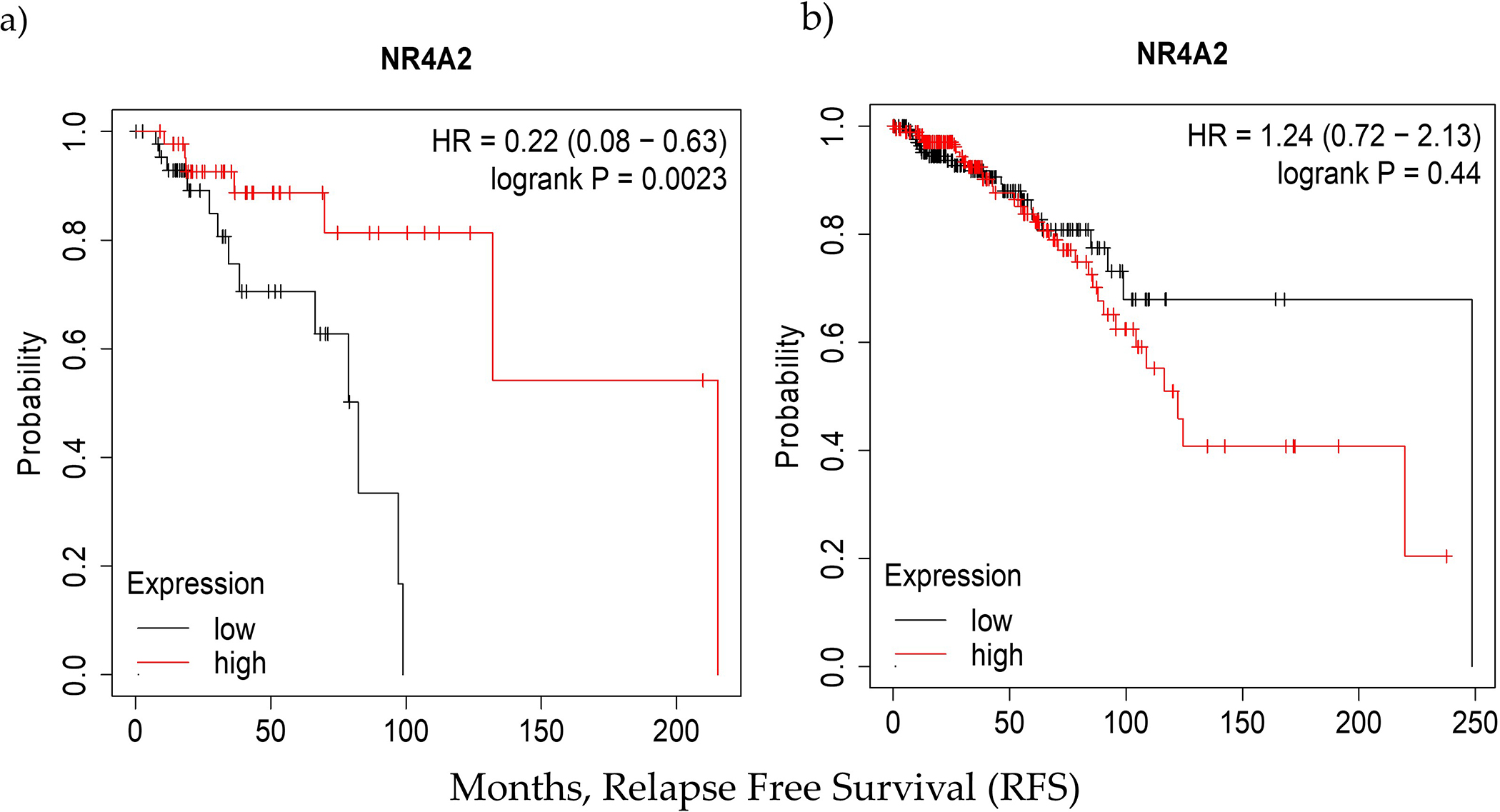
KM plots indicating the significant variation in the RFS values in (**a**) Black and (**b**) White racial groups as function of NURR1 expression in upper and lower quartile. In Black population low expression of NURR1 expression is associated with poor RFS (82.3 months) rate while in white it resulted in relatively higher RFS (248.5 months).

We compared the predictive value of NURR1 in Black and White patients using TCGA data (PanCancer Atlas) through KMPLOT. Specifically, we compared the overall survival of patients with upper vs. lower quartile expression of NURR1 as derived from mRNA next generation sequencing (NGS). Interestingly, NURR1 was associated with greater overall survival of Black, but not of White BCa patients through 250 months (Figure 3). For Black patients, the hazard ratio (HR) was reduced to 0.22 (0.08–0.63) with logrank *p* of 0.0023 for patients expressing high vs. low NURR1. In White patients, the hazard ratio (1.24) was increased, though it did not reach statistical significance (0.72–2.13) with logrank *p* of 0.44. In White patients, the median survival for the low expression cohort was 248.5 months, versus 122.3 for the high expression group. For Black patients, the values were drastically different, with the low expression cohort experiencing a median survival rate of only 82.3 months, and the high expression cohort 215.2 months. With the median RFS for all patients being 127.3 and 129.7 for Black and White patients respectively, it is evident that the loss of NURR1 expression is a negative biomarker within the cohort of Black patients without having a similar significance for White BCa patients. Notably, the overall survival of Black women with high expression of NURR1 was greater than the median OS of both Black and White cohorts.

**Figure 4.**
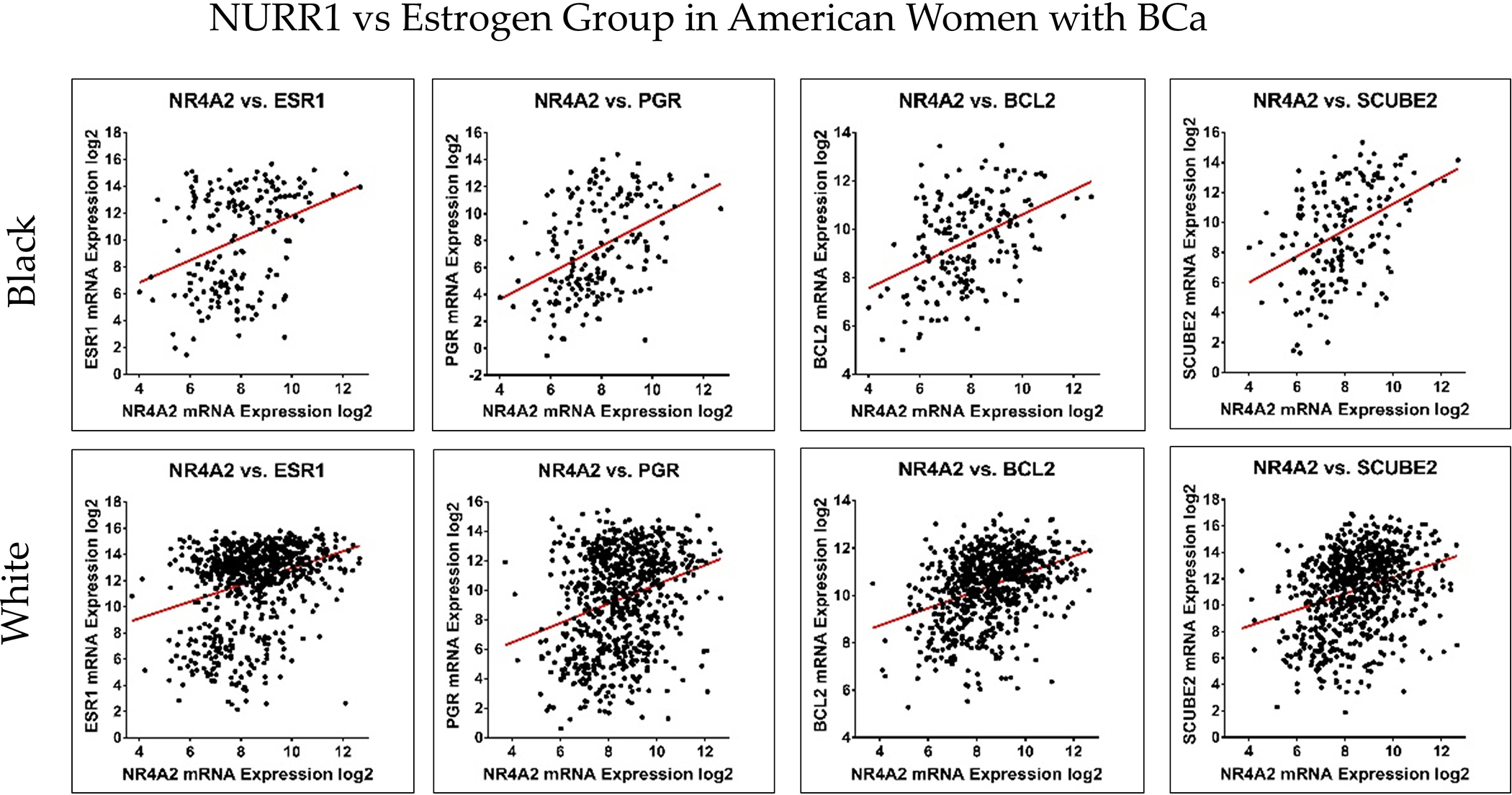
NURR1 expression is positively correlated with Oncotype DX estrogen signaling group. The correlation trend of NURR1 expression with estrogen markers was found to be positive in both Black (**Top row**) and White (**Bottom row**) BCa women. The X and *Y*-axis represent the log2 of mRNA expression of RSEM batch normalized from illumina HiSeq-RNASeqV2.

**Figure 5.**
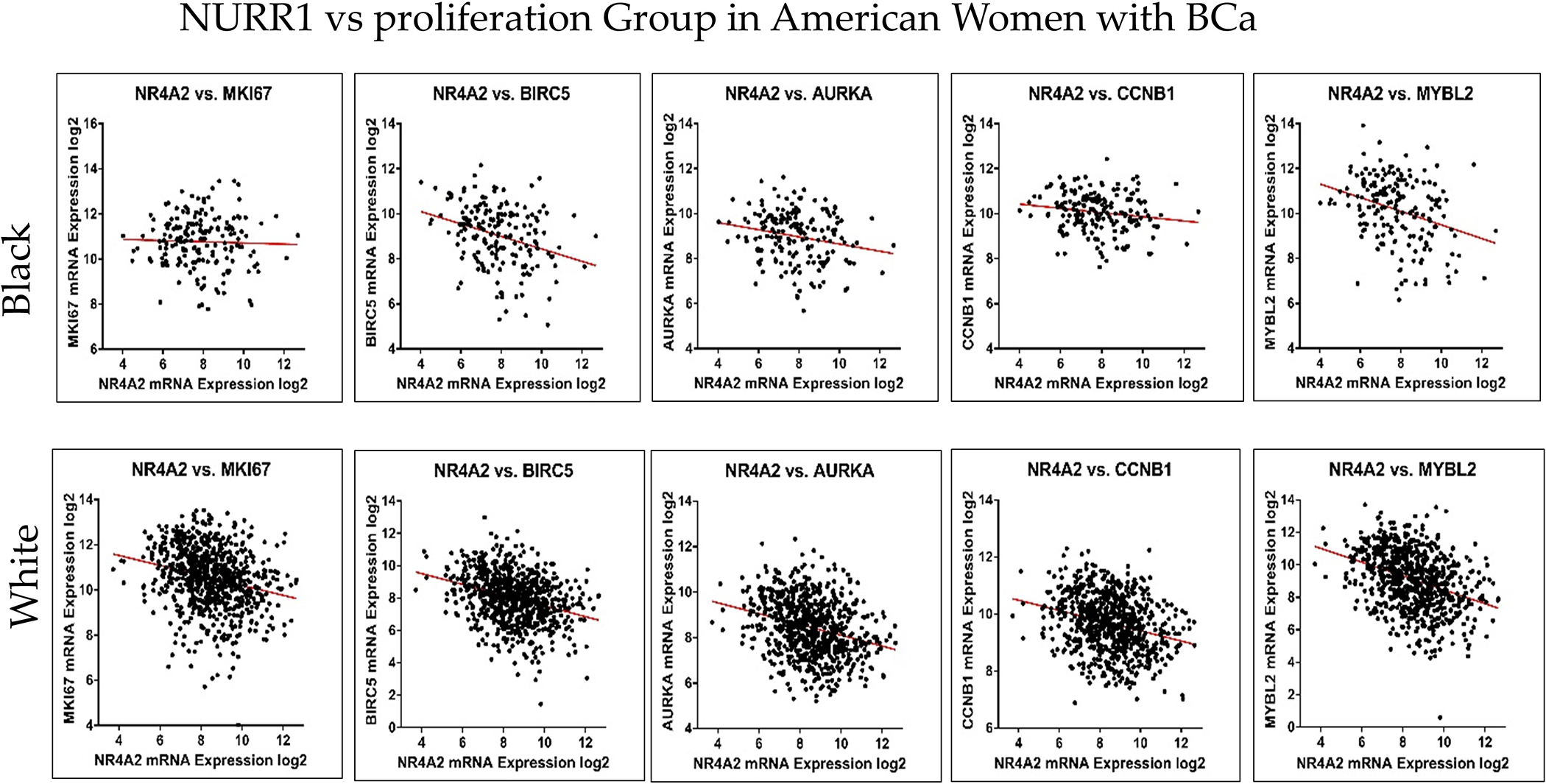
NURR1 expression is negatively correlated with Oncotype DX proliferation group. The correlation trend of NURR1 with proliferation markers was found to be negative in both Black (**Top row**) and White (**Bottom row**) BCa women. The X and *Y*-axis represent the log2 of mRNA expression of RSEM batch normalized from illumina HiSeq-RNASeqV2.

Patient risk stratification according to gene expression type has been utilized for treatment decision-making processes in BCa. Since NURR1 appears to be a biomarker for RFS in Black BCa patients, we performed co-expression analysis of NURR1 with the expression of the Oncotype DX gene expression panel to determine if NURR1 expression held similar predictive value as Oncotype Dx component genes. ESR1, SCUBE2, BCL2, and PGR expression were all strongly correlated with NURR1 expression (Figure 4, Table 2). Conversely, CCNB1, CTSV, AURKA, and MYBL2 in proliferation group showed strong negative correlation to NURR1 expression (Figure 5, Table 2). To determine if NURR1 expression showed comparable prognostic value as existing biomarkers for BCa survival among Black patients, we compared the overall survival of Black patients stratified by upper and lower quartiles of expression with regard to NURR1, ESR1, or the oncotype DX expression panel.

**Figure 6.**
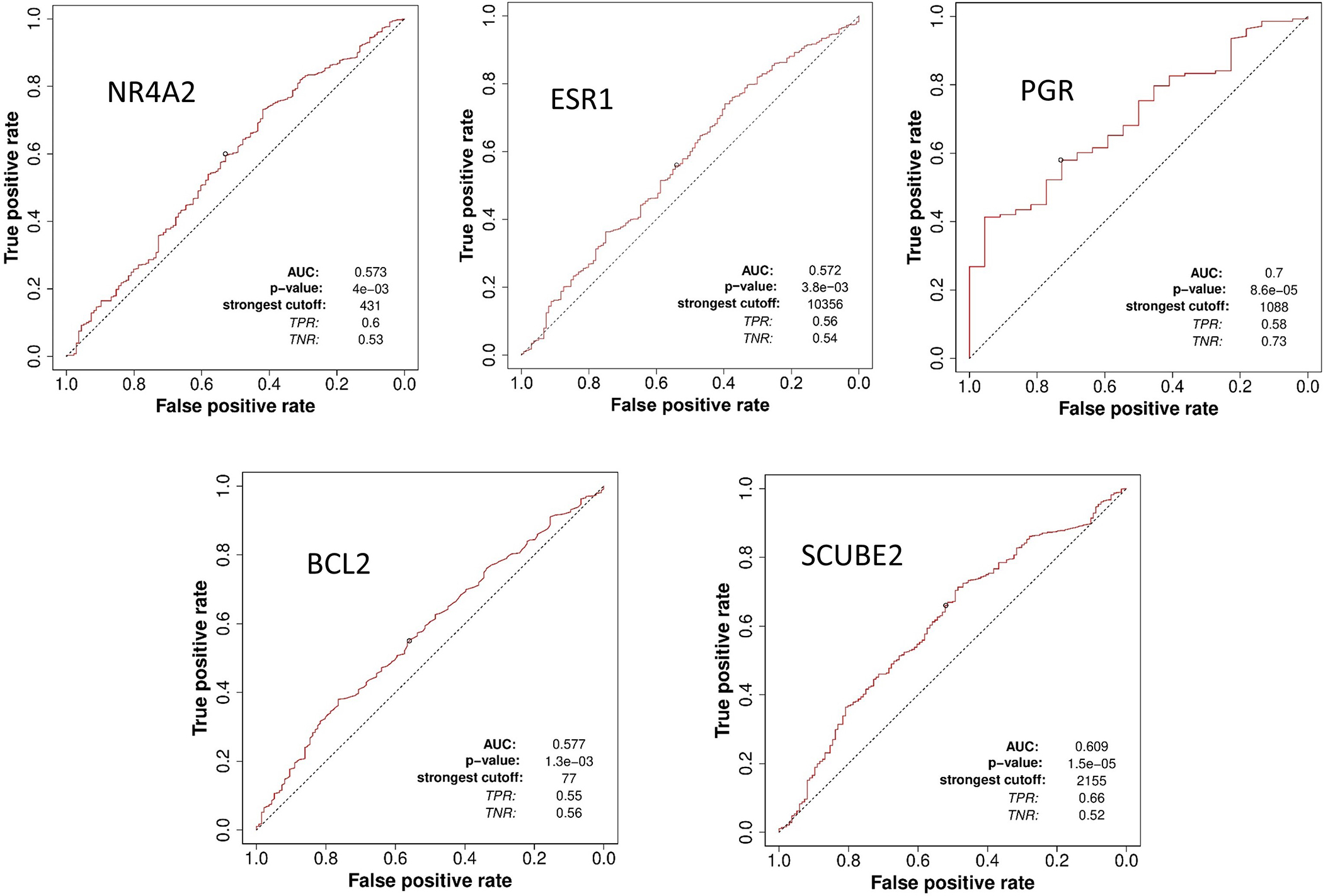
ROC plotter predictive biomarker analysis suggests NURR1 can be potential breast cancer biomarker. The AUC of NURR1 expression in total population with 5-year relapse free survival with endocrine therapy in comparison to other breast cancer biomarkers was found to be similar especially with ESR1, BCL2, and SCUBE2.

The receiver operator characteristic (ROC) plotter tool is an online tool used to assess the predictive characteristics of specific genes with clinical outcomes in several cancers, including BCa [23]. ROC plotter’s validation for predictive cancer biomarkers analysis of NURR1 with 5-year relapse-free survival in the area under the curve (AUC) of 0.573 (*p* value 4 × 10^−3^) indicating a potential biomarker (Figure 6) [23]. We have compared the AUC of NURR1 with other established breast cancer biomarkers in the estrogen signaling group of Oncotype DX since NURR1 expression is positively correlated with this group (Figure 4). The AUC of other breast cancer makers are as follows: ESR1 0.572 (*p* value 3.8 × 10^−3^), PGR 0.7 (*p* value 8.6 × 10^−5^), BCL2 0.577 (*p* value 1.3 × 10^−3^), SCUBE2 0.609 (*p* value 1.5 × 10^−5^) (Figure 6). The AUC of NURR1 is found to be similar to ESR1, BCL2, and SCUBE2. These findings reinforce our assertion that NURR1 expression is associated with sensitivity to endocrine therapy can be potential biomarker.

## 4. Discussion

The underrepresentation of people of color in various clinical trials and the mismatch between clinical trial representation with the real-world population has been documented by various studies [25]. Multiple socioeconomic/cultural factors such as educational attainment, time, costs, religious/spiritual beliefs, transportation, and mistrust act as obstacles for the lack of diversity in clinical trials [26]. Moreover, the infamous historical examples of exploitation such as the Tuskegee syphilis study [27] and Henrietta Lacks cell line derivation [28] further fuel the skepticism of the clinical research among African American community and may also contribute to the low participation. Loree et al., reviewed 230 reported clinical trials that led to FDA cancer drug approvals from the period of July 2008 to June 2018 [29]. They found that only 145 (63%) trials have mentioned race and the overall proportion of Blacks’ participation was only 4.5% despite representing 13.6% of the US population [29]. Furthermore, a slight decrease in the relative proportion of Black patients participation was noticed from 3.6% to 2.9% (2008–2013 vs. 2013–2018) [29]. The drug approvals resulting from non-inclusive clinical trial designs do not reflect significant heterogeneity in the US population, and as such these trials cannot accurately represent real world diversity in disease progression, pharmacological responses, and adverse drug reactions. Therefore, the increase participation of Black and other minority groups in clinical trials would contribute to more reliable clinical trials results.

A recent study conducted on 1332 women (including 666 Black) found that Black women had larger tumor sizes, increased nodal disease, and higher percentages of triple negative subtype with poor disease-free survival and overall survival [30]. The identification and establishment of a novel prognostic basal-like cancer breast cancer marker has tremendous potential in the detection and improvement of the survival rate in Black women who often get this type of breast cancer. Some of the notable biomarkers are VEGF, EGFR, Ki67, TP53, TOP2A, PRAP, and BRCA [31]. However, most studies have focused on the expression of these biomarkers in basal-like breast cancer, despite the fact that Black patients with Luminal A BCa also experience disparity in RFS as compared to their White counterparts. As such, it is necessary to identify additional risk factors beyond the existing phenotypic descriptors that can function as a prognostic indicator for BCa patients with luminal A disease. Such a biomarker would have an outsized impact on Black patients, given the continued disparity in survival of patients with this phenotype.

In our previous studies we have shown that the orphan nuclear receptor NURR1 is most highly expressed in normal breast epithelium, is repressed transformed breast epithelium [18]. In BCa, we reported that ER was more likely co-expressed in NURR1(+) than in NURR1(−) tissue, and that NURR1 expression was associated with greater RFS in systemically treated BCa patients. Our current studies interrogating NGS data are in concordance with our previous studies. Here we show that NURR1 expression is repressed in basal-like cancer, and commonly expressed in Luminal A tumors. Interestingly, we also observed that even though Black and White patients with basal-like cancer exhibited low expression of NURR1, we found that the mean NURR1 expression between Black and White patients was significantly different with Black patients’ tumors having much lower NURR1 expression.

The oncotype DX assay’s recurrence score (RS) is dependent on the 16 cancer-related gene expression levels [13]. These genes are classified into various groups where each group having either positive or negative coefficient that influences the overall RS [13]. There are four groups, namely proliferation (5 genes), estrogen (4 genes), invasion (2 genes), and HER2 (2 genes), while the remaining 3 genes GSTM1, BAG1, and CD68 each have their individual coefficients (Table 1) [13]. The *RS* is the sum of all these scores multiplied to their coefficients as shown in the equation below.


(1) RS=(1.04)(Proliferationgroup)+(0.47)(HER2group)+(0.1)(Invasiongroup)+(0.05)(CD68)+(−0.34)(ERgroup)+(−0.08)(GSTM1)+(−0.07)(BAG1)

**Figure 7.**
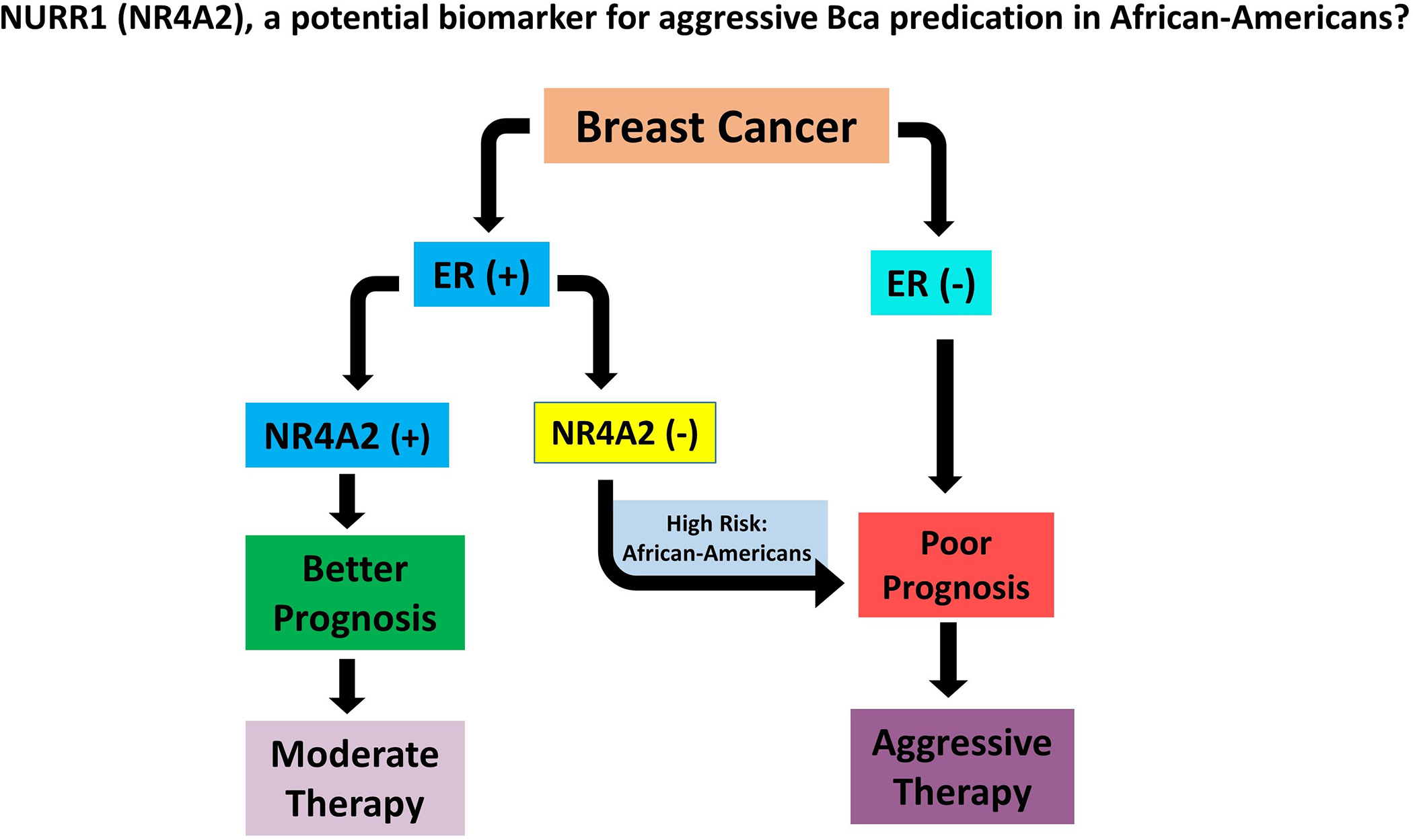
ER(+) breast cancer with low expression of NURR1(NR4A2) could result in poor prognosis leading to aggressive chemotherapy. Patients with positive expression of NURR1 in ER (+) BCa respond favorably to moderate therapy. African-American women with ER(+) BCa showed low levels of NURR1 and thus at high risk of poor survivorship.

The RS equation indicates that the increased expression of proliferation and HER2 groups majorly contribute to the raise in RS whereas increase in ER *group* gene expression results in lower RS values [13]. The RS scale is on 0–100 where the RS<18 is considered as “low risk”, the RS>/=31 is “high risk”, while the score in between 18 and 31 is “intermediate risk” [13]. We found that the expression of NURR1 is positively correlated with genes in estrogen group (Figure 4) while negative correlation with proliferation group is observed (Figure 5). Although the role of NURR1 in breast cancer is not well-understood yet, this positive correlation with ERgroup and negative correlation with proliferation group indicates that NURR1’s expression can be used to predict the poor outcomes of LumA subtype cases and help to decide whether an aggressive or a moderate therapy is necessitated. It is evident from our data that Black patients tend to have poor survival rates with low expression of NURR1 than the White patients in luminal A BCa. Based on this, we hypothesize that the lack of NURR1 results in the poor survival outcome in luminal A BCa cases (Figure 7). Regardless of race, we think that if the NURR1 expression is low enough it could result in poor survival outcome; however, the overall expression in majority of Black patients is too low, resulting in poor prognosis (Figure 7). It is our assertion that NURR1 expression may be an effective prognostic indicator for therapeutic efficacy among patients with luminal A BCa. With our finding that NURR1 (−) BCa occurs disproportionately among Black BCa patients, the implementation of this biomarker clinically may have a significant impact on survivorship disparities.

## 5. Conclusions

Our studies suggest that NURR1 is differentially expressed at higher levels in less aggressive BCa phenotypes, and that its expression is likely a marker for endocrine sensitivity among patients with luminal A BCa. Additionally, we show that NURR1 is expressed at lower levels among Black women with BCa, and that this may contribute to disparities associated with BCa survivorship. These findings suggest that NURR1 may function as a biomarker for favorable outcome in BCa and may be a feasible pharmacologic target in BCa. Furthermore, this may yield insight into the factors associated with continued racial disparities in breast cancer survival, which persist even among Black patients with so-called favorable BCa phenotypes.

## Figures and Tables

**Table 1.** Oncotype DX 21 gene classification into various groups.

Proliferation Group	Estrogen Group	Invasion Group	HER-2 Group	Other Genes	Reference Genes
Ki-67	ER	Stromelysin 3	GRB7	GSTM1	Beta-Actin
STK15	PR	Cathepsin L2	HER2	BAG1	GAPDH
Survivin	Bcl2			CD68	RPLP0
Cyclin B1	SCUBE2				GUS
MYBL2					TFRC

**Table 2.** The correlation of NURR1 expression with respect to expression of genes in Oncotype DX panel in Black and White breast cancer women.

Group	Gene	Spearman’s Correlation					
		Black Women			White Women		
		Coefficient	*p*-value	R^2^	Coefficient	*p*-value	R^2^
Estrogen	ESR1	0.31	2.694 × 10^−5^	0.11	0.29	3.31 × 10^−16^	0.1
	PGR	0.43	2.09 × 10^−9^	0.18	0.28	6.10 × 10^−15^	0.09
	Bcl2	0.39	5.61 × 10^−8^	0.18	0.37	1.94 × 10^−25^	0.14
	SCUBE2	0.42	4.52 × 10^−9^	0.18	0.32	4.89 × 10^−18^	0.1
Proliferation	MKi-67	−0.02	0.810	0	−0.27	1.95 × 10^−14^	0.06
	STK15	−0.2	6.760 × 10^−3^	0.05	−0.3	1.04 × 10^−16^	0.08
	Survivin	−0.31	2.747 × 10^−5^	0.1	−0.33	1.02 × 10^−20^	0.1
	Cyclin B1	−0.16	0.0315	0.03	−0.27	3.37 × 10^−14^	0.07
	MYBL2	−0.29	5.675 × 10^−5^	0.1	−0.37	1.83 × 10^−25^	0.12
Invasion	Stromelysin 3	0.03	0.661	0	−0.15	5.914 × 10^−5^	0.03
	Cathepsin L2	−0.20	7.787 × 10^−3^	0.04	−0.2	2.08 × 10^−8^	0.05
HER-2	GRB7	−0.08	0.308	0.01	0.02	0.513	0
	HER2	0.1	0.193	0	0.11	2.577 × 10^−3^	0
Other	GSTM1	0.05	0.512	0	0.08	0.0362	0
	BAG1	0.1	0.180	0.02	0.26	1.34 × 10^−12^	0.07
	CD68	−0.02	0.808	0	−0.07	0.0662	0.01

## Data Availability

All data sets used in this study are publicly available at https://www.cbioportal.org/, https://kmplot.com/analysis/index.php?p=service&cancer=pancancer_rnaseq (all accessed on 13 October 2022) and ROC Plotter—Online ROC analysis.
